# Crystallization of Covalent Organic Frameworks for Gas Storage Applications

**DOI:** 10.3390/molecules22071149

**Published:** 2017-07-10

**Authors:** Lijuan Zhu, Yue-Biao Zhang

**Affiliations:** School of Physical Science and Technology, ShanghaiTech University, Shanghai 201210, China; zhulj@shanghaitech.edu.cn

**Keywords:** covalent organic framework, crystallization, dynamic covalent chemistry, hydrogen storage, methane storage, CO_2_ capture

## Abstract

Covalent organic frameworks (COFs) have emerged as a new class of crystalline porous materials prepared by integrating organic molecular building blocks into predetermined network structures entirely through strong covalent bonds. The consequently encountered “crystallization problem” has been conquered by dynamic covalent chemistry in syntheses and reticular chemistry in materials design. In this contribution, we have reviewed the progress in the crystallization of COF materials and their hydrogen, methane and carbon dioxide gas storage properties for clean energy applications.

## 1. Introduction

In recent decades interest in organic materials with permanent nanometer scale pores has grown very quickly due to their specific properties and broad applications in gas storage, gas separation, drug delivery, energy conversion, catalysis, and optoelectronics. Scientists have designed and constructed a large number of porous materials, such as zeolites, metal-organic frameworks (MOFs), mesoporous silica, organosilica, microporous polymers, porous carbons, covalent organic frameworks (COFs) and organic molecular cages. These porous materials can be designed with variation of components, functionalities, pore metrics and skeletons. During the past decades, construction of persistent organic porous materials via covalent bonds is attracting increasing interest due to their unique stability and advanced applications. However, the wide pore-size-distribution and disordered extended structures of these amorphous networks restrict their further applications. Therefore, COFs as a class of covalent porous crystalline polymer in which the organic building blocks are covalently integrated to form extended ordered crystal structures, makes them different from the amorphous porous organic polymers [1,2,3,4,5,6]. The first synthetic challenge to make COFs, which was even thought impossible due to the difficulty of linking organic building blocks into crystalline extended structures entirely through strong covalent bonds, is the so-called “crystallization problem”, the lack of solubility and reversibility for correcting errors during crystallization. Such difficulties were first conquered by the judicious choice of reversible condensation reactions where water is the byproduct, whose retention can be further used to control the direction of reactions for good reversibility. Meanwhile, the bond energy is also a key parameter for the ease of bond breaking and reformation during crystallization. It is worth mentioning that it is easy to used weaker covalent bonds to achieve higher crystalline products, but the framework’s robustness necessary to achieve practical functions, such as permanent porosity and structure integrity in applications, would then be compromised. Obviously, there is a trade-off between crystallinity and robustness in making COF in the current state of the art. We do believe, however, that this situation will be resolved in the near future by knowing how to control the reversibility and the precision of molecular design.

## 2. Dynamic Covalent Chemistry of COFs

**Scheme 1:**
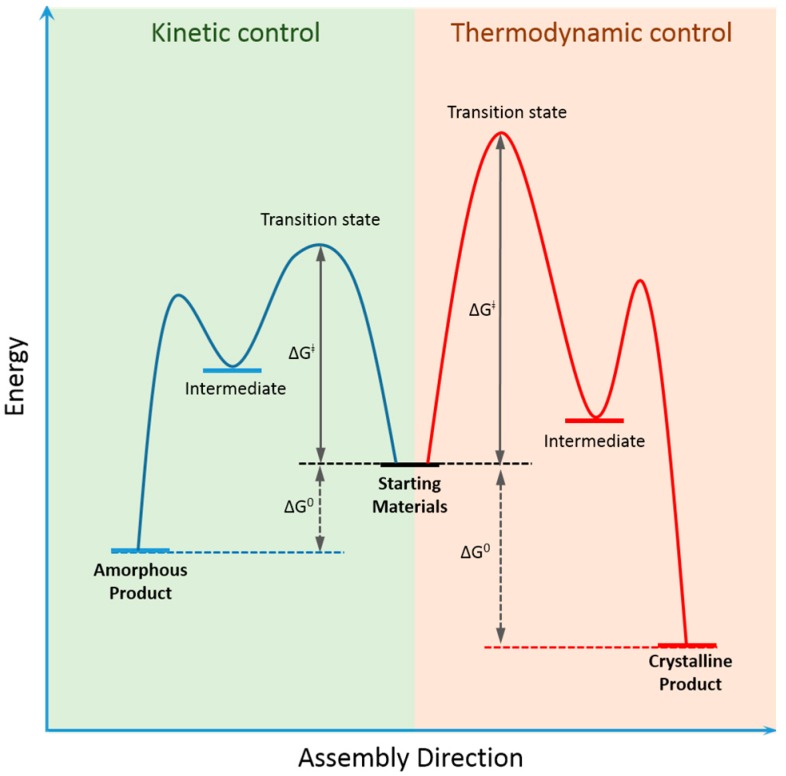
Thermodynamic control in the dynamic covalent chemistry of COFs.

In order to achieve higher reversibility for efficient error correction during crystallization, dynamic covalent chemistry (DCC) has been well applied in the construction of COFs. To stitch discrete molecular building blocks into periodic networks, DCC enables strong covalent bond formation accompanied by the assembly of molecular building blocks into predetermined spatial arrangements [7,8]. Considering the possible spatial arrangements of the moiety formed in the reaction, there are multiple products in the equilibrium with various degrees of crystallinity. Such reversible assembly of molecular components can proceed along kinetic or thermodynamic pathways (Scheme 1). Due to the lower barrier of activation (Δ*G*^‡^), kinetic intermediates dominate initially due to a fast rate of formation the kinetic products, which can be trapped during quenching of the reaction and form amorphous products with the gain of entropy from their randomness of arrangement, meanwhile the thermodynamic products have the lowest overall Gibb’s free energy (Δ*G*°), so that given the opportunity the reaction will re-equilibrate towards the global minimum to form the most stable and probably crystalline products. It is also possible that the kinetic products can also be transformed into thermodynamic products by using certain thermo-treatments for recrystallization. It is common in COF chemistry that amorphous products are initially obtained until the synthetic conditions are optimized by varying the crystallization times, reaction temperatures, reaction solvents, and catalysts (such as acids, bases, and metal salts acting as Lewis acids). The discovery process may change the activation energy of thermodynamic products and their reaction rates to accelerate the crystallization of COFs. On the other hand, rational design of building blocks may also increase the correctness of the assembly of molecular components, thus enhance the crystallinity of the obtained products. It is generally believed that 2D COFs are easier to form having less probability of resulting in networks than the 3D ones with more structural diversity.

**Figure 1:**
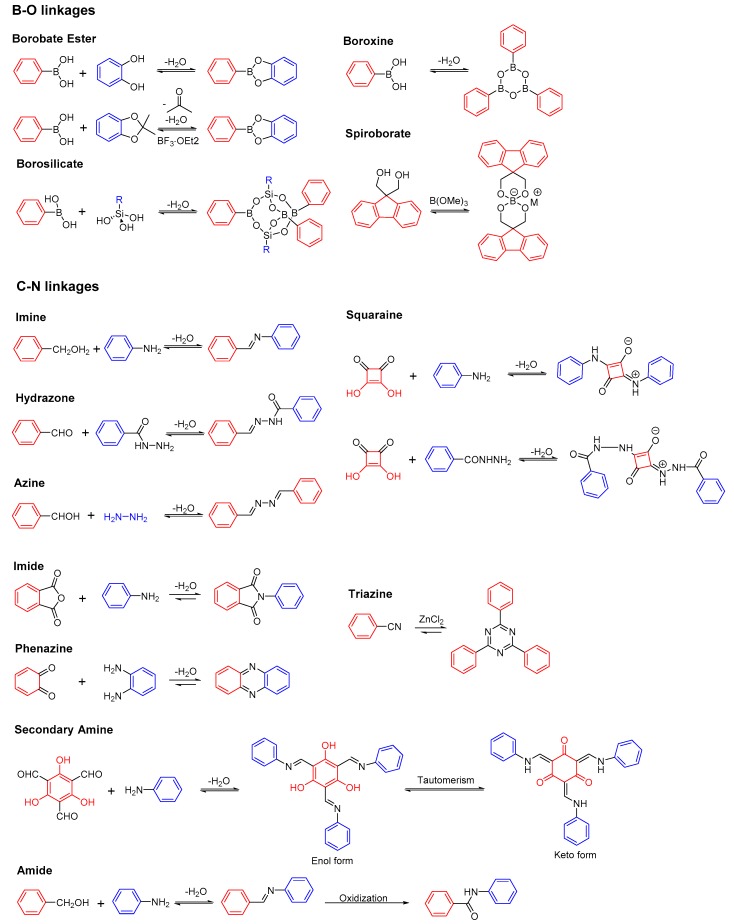
Several reversible reactions used for construction of COF materials. Dynamic reversible reactions are essential for the formation of crystal covalent organic frameworks (COFs). These reversible reactions for the formation of COFs are boron-containing linkages (for example, boroxine, boronate ester, borazine and spirobrate), nitrogen-containing linkages (for example, imine, hydrazone, azine, squaraine, phenazine, triazine, imide and azodioxy), and a double-stage linkage containing the boronate ester linkage and the imine linkage in one COF via a one-pot reaction.

From the chemistry viewpoint, the difficulty of constructing COF materials can be classified by the difference of bond energy of the linkages between the molecular building blocks (Figure 1). It is easier for weak covalent bonds to break and re-form to correct assembly errors, such as the reversible covalent bond have enabled to make products with large single crystals [9]. However, the lack of robustness prevents their permanent porosity for further applications. For stronger covalent bonds, their reversibility during the reactions are highly dependent on the reaction conditions, which can perform both crystallinity and robustness after isolation from the system.

### 2.1. B-O Linkages

Featuring light-weight compositions and high crystallinity, boron-based COFs have been intensively studied including understanding their crystallization mechanism, to the designing of new topologies, achieving high surface area, and the utilization in gas storage applications. The successful construction of such crystalline materials benefited from the decent reversibility of the B-O linkages, including boroxine (-B_3_O_3_), dioxaborole (-BO_2_C_2_), and spiroborate (-O_4_B¯).

#### 2.1.1. Boroxine-Linked COFs

**Figure 2:**
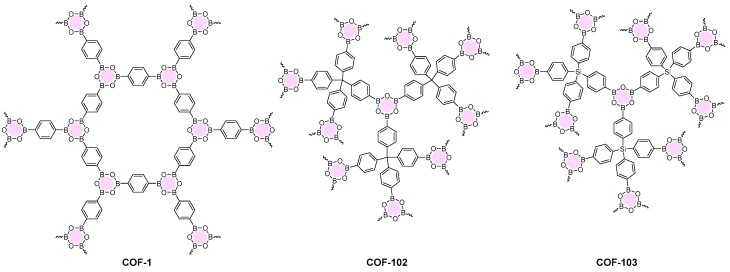
Chemical structures of classic boron-containing COFs (COF-1, COF-102, COF-103) with boroxine connections.

The emergence of COF chemistry was triggered by Yaghi and co-workers’ seminal work on the self-condensation of aromatic polyboronic acids (Figure 2) [10]. The successful crystallization of these materials was attributed to the implementation of a closed reaction system to sustain the availability of water for maintaining reversible conditions conducive to crystal growth. The solvents used and their mixture are a handle to control the diffusion of building blocks into the crystallization mother liquor. The crystallization was intentionally processed slowly and terminated after sufficient time had elapsed.

The choice of solvent and temperature to maximize the error correction associated with the boroxine ring-forming reactions can optimize the crystallinity of the boroxine-based COFs. COF-1 with two-dimensional (2D) architectures, was prepared by the self-condensation of 1,4-phenylenediboronic acid (BDBA) under solvothermal conditions, to produce an extended staggered layered structure with hexagonal pores of 15 Å diameter and a BET surface area of 711 m^2^·g^−1^. Yaghi and co-workers also synthesized three-dimensional (3D) COFs, COF-102 and COF-103 by self-condensation of tetrahedral (3D-*T*_d_) nodes, tetra(4-dihydroxyborylphenyl)methane (TBPM) and its silane analog (TBPS), respectively (Figure 2) [11]. These two COFs are the crystal porous organic frameworks with high surface areas (3472 m^2^ g^−1^ for COF-102 and 4210 m^2^ g^−1^ for COF-103) and pore size distributions of 11.5 Å (COF-102) and 12.5 Å (COF-103).

Microwave heating can accelerate the reaction times and obtain much cleaner COFs with high yields. Cooper and co-workers demonstrated the synthesis and purification of COF-5 and COF-102 by microwave heating. The microwave synthetic method reported by Cooper and co-workers was 200 times faster than solvothermal methods reported by Yaghi et al. without changing the physical properties of COFs [12].

#### 2.1.2. Dioxaborole-Linked COFs

**Figure 3:**
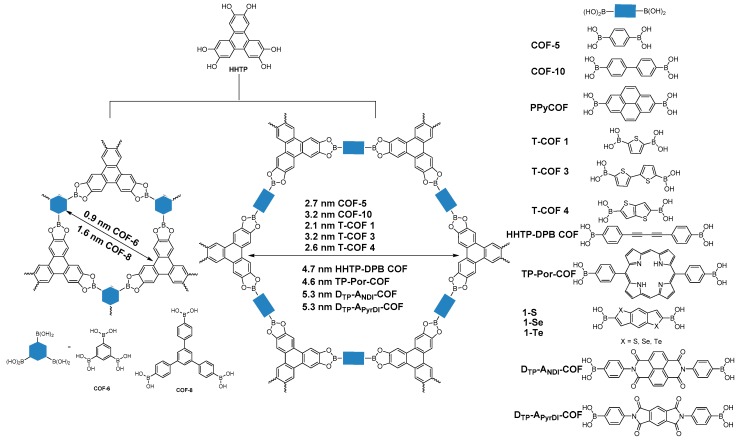
Construction of dioxaborole-linked COFs via the cross-condensation of triangular HHTP with ditopic or tritopic boronic acids to form 2D honeycomb layer structures.

The cross-condensation of polybornic acids with catechols opened the way to stich different building blocks into one framework (Figure 3). The popular utilization of 2,3,6,7,10,11-hexahydroxytriphenylene (HHTP) as a triangular building block with ditopic, tirtopic and tetratopic boronic acids leads to 2D and 3D networks (Figure 3) [10,11,13]. With a sealed reaction system, COF-5, -6, -8 and -10 were successfully prepared in the cocktail solvent system by mixing mesistylene and 1,4-dioxane for 3–5 days at 85–100 °C [10,13]. A similar COF with the same HHTP as co-linker can be also prepared by stirring under an inert atmosphere [14].

By using the tetrahydroxybenzene and its derivatives, 2D COFs can be prepared by stitching tritopic boronic acids together. The reactions were conducted in the mixture of tetrahydrofuran (THF) and methanol (MeOH) by stirring under an inert atmosphere [15,16].

**Figure 4:**
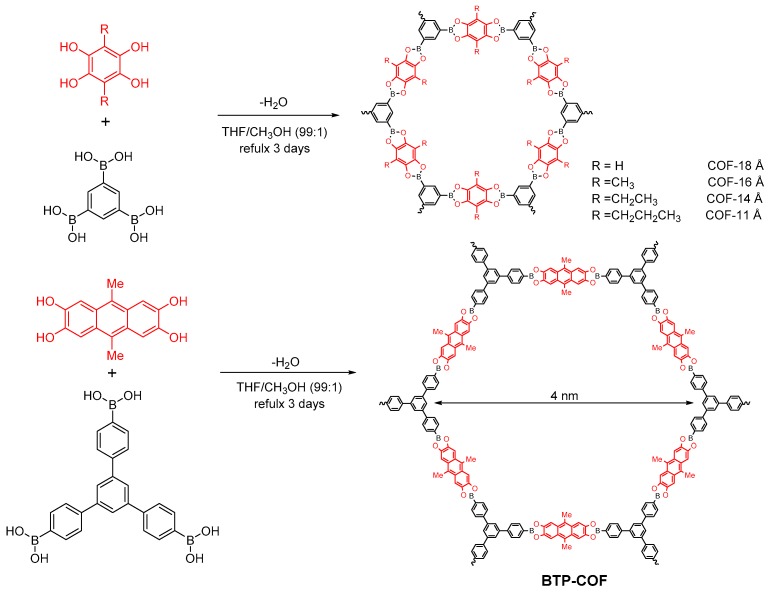
Construction of dioxaborole-linked COFs via the cross-condensation of ditopic catechol-based linkers with tritopic boronic acids to form the 2D honeycomb layer structures. The pore sizes can be modulated by the decorated functionalities of the ditopic linkers, and expanded by elongating both the boronic acid and catechol linkers.

Boronate ester linked COFs have high crystallinity, but low hydrolytic stability and chemical stability because of the reversibility of the reactions, which leads to decomposition upon exposure to water or acid. This disadvantage greatly limits their applications. Alkylation in the pores of COFs improves the stability by decreasing the hydrolysis rate of the connected linkage. Lavigne and co-workers were the first to incorporate alkyl groups into the channel walls during the synthesis of the boronate ester linked COFs to increase the stability of COFs, as well as to fine tune the pore sizes [16]. The presence of alkyl chains in the channels control the pore size from 1.8 nm to 1.1 nm (Figure 4), which were observed for the series of COF-18 Å (no alkyl group), COF-16 Å (methyl), COF-14 Å (ethyl), and COF-11 Å (propyl). Meanwhile, the surface area decreased from 1263 to 105 m^2^·g^−1^ and the pore volume decreased from 0.69 to 0.052 cm^3^·g^−1^, as the chain length increased. Interestingly, modification of the pore interior with increasingly larger alkyl groups causes a decline in nitrogen uptake but an increase in the molar amount of hydrogen adsorbed. Once submersion in aqueous media, the porosity of alkylated COFs decreased by about 25%, while the non-alkylated COFs were almost completely hydrolyzed, losing virtually all porosity. In addition, the degree of crystallinity decreased by about 40% for alkylated COFs and 95% for non-alkylated COFs. By using microwave synthesis, Bein et al. extended the framework into materials having large 4 nm openings (Figure 4), featuring one of the largest pores in crystalline materials at this time [17].

**Figure 5:**
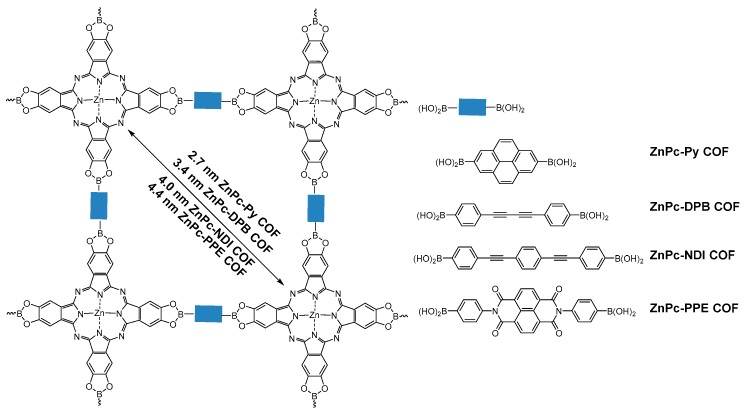
Construction of dioxaborole-linked COFs via the cross-condensation of ditopic catechol-based linkers with tritopic boronic acids to form 2D honeycomb layer structures. The pore sizes can be modulated by the decorated functionalities of the ditopic linkers, and expanded by elongating both the boronic acid and catechol linkers.

By using octahydroxyphthalocyanine and its metalated version, the Dichtel group was able to expand the pore size of the ZnPc lattice from 2.7 to 4.4 nm (Figure 5) [18]. The Dichtel group also reported the selective and patterned growth of a 2D phthalocyanine COF on single-layer graphene. This provides a promising pathway to direct the growth of COF films [19].

**Figure 6:**
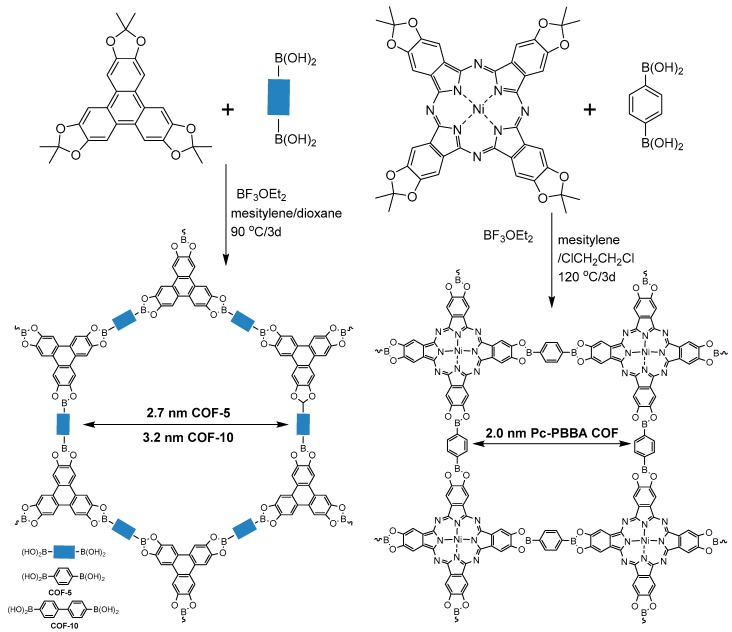
Synthesis of dioxaborole-linked COFs starting from protected catechol-based linkers with boronic acids for improved solubility and anti-oxidation in comparison with pristine catechol linkers.

To avoid the oxidation and insolubility of polycatechols, protected catechols were proposed as starting materials for making COFs which can be deprotected in-situ under catalysis by a Lewis acid such as BF_3_·OEt_2_ [20]. With this strategy, triangular HHTP and square phthalocyanine as well as its metalated derivatives have been successfully incorporated into boronate ester-linked COFs (Figure 6). Mechanistic studies show that before adding the Lewis acid, the boronic acids self-condensate reversibly to form boroxine-linked products and H_2_O. The addition of BF_3_·OEt_2_ catalyzes acetonide hydrolysis of protected catechols, and the resulting catechol will rapidly condense with the boronic acids to form the boronate-linked COFs [21]. Once the free boronic acids are consumed, the formed products will hydrolyze and release more boronic acids, which will produce more boronate-linked COFs.

**Figure 7:**
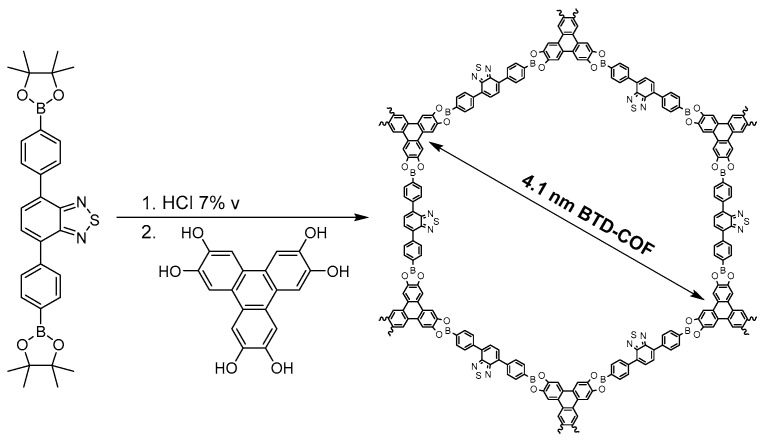
Synthesis of the dioxaborole-linked COF by in-situ deprotection of boronic pinacol ester followed with the addition of HHTP via microwave heating method.

To accelerate the synthesis of highly crystalline COF material, a two-step microwave synthesis has been proposed for protected boronic acids as starting materials with the addition of HHTP (Figure 7). The resulting product proved to be highly crystalline and possess large pore openings. Control experiments didn’t yield COF via the one-pot reaction. This work also provided the first high-resolution TEM image of a COF showing the pores clearly [22].

**Figure 8:**
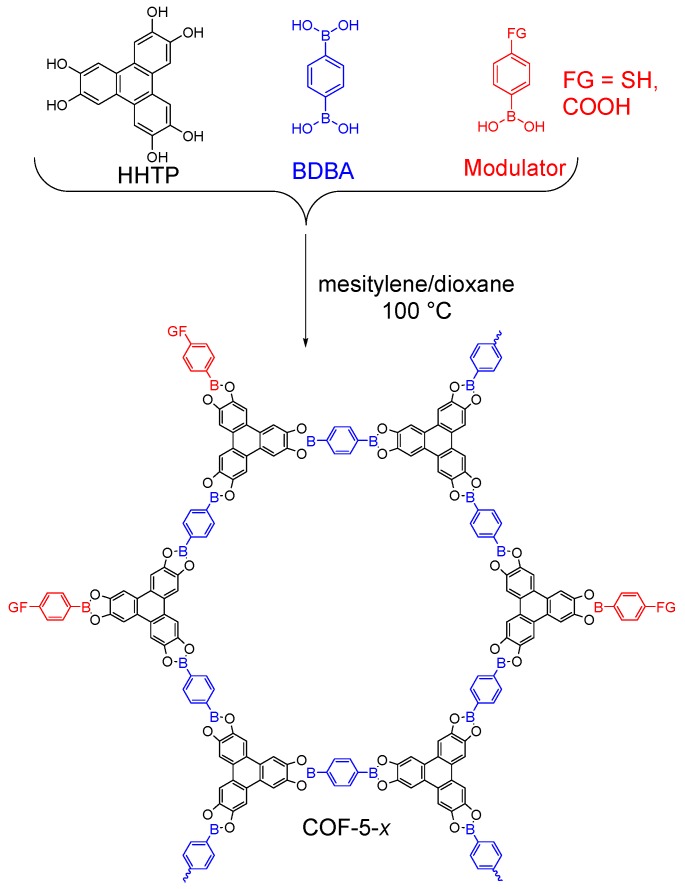
The modulation approach to synthesize COF-5-*X* via the cross-condensation of HHTP and 1,4-diboronic acid combining with terminal boronic acids with various functional groups to achieve higher crystallinity and external functionalities.

A modulation approach has been developed to control the structure and crystallinity of COF materials, introducing a modulator in the synthesis that can compete with one of the building blocks during the solvothermal COF growth to form highly crystalline frameworks with large domain size and very high porosity. Bein and co-workers used monoboronic acids as modulators in the solvothermal synthesis of the archetypical COF-5 to optimize the crystallinity, domain size, and porosity of 2D COFs (Figure 8). Moreover, the addition of the monoboronic acids also provides a potential construction of functional crystalline COF materials [23].

#### 2.1.3. Spiroborate-Linked COFs

**Figure 9:**
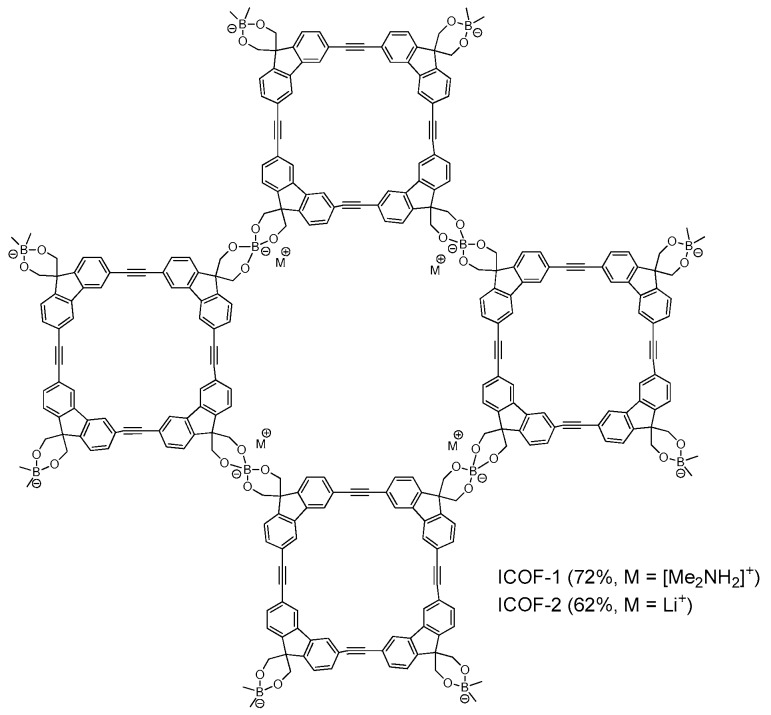
Construction of spiroborate-linked COFs featuring anionic skeletons with various counter ions for high ionic conductivity.

Researchers have shown great interest in exploring new methods to enhance the chemical stability of boron-containing COFs. Recently, the condensation of diols with trialkyl borate in the presence of basic catalysts has been explored for the synthesis of spiroborate-linked ionic COFs in which the negatively charged borons are located on the edges with different cations as counter ions. Zhang and co-workers [24] constructed a novel type of spiroborate-linked ionic covalent organic framework (ICOF, Figure 9), which contains sp^3^ hybridized boron anionic centers and tunable countercations (lithium or dimethylammonium). The synthesized ICOFs (ICOF-1 and ICOF-2) show good thermal stabilities and excellent resistance to hydrolysis, remaining nearly intact when immersed in water or basic solution for up to two days. The SEM images support the single-crystalline morphology of ICOF-1 and ICOF-2. The PXRD results also show multiple sharp peaks, which indicate the orderliness structures in the framework. However, the crystal structures of ICOFs have not been characterized. These ICOFs also have high BET surface areas up to 1259 m^2^·g^−1^ and adsorb a significant amount of H_2_ (up to 3.11 wt %, 77 K, 1 bar) and CH_4_ (up to 4.62 wt %, 273 K, 1 bar). The existence of permanently immobilized ion centers in ICOFs enables the transportation of lithium ions with room-temperature lithium-ion conductivity of 3.05 × 10^−5^ S·Lcm^−1^ and an average Li^+^ transference number value of 0.80 ± 0.02.

### 2.2. C-N Linkages

#### 2.2.1. Imine-Linked COFs

**Figure 10:**
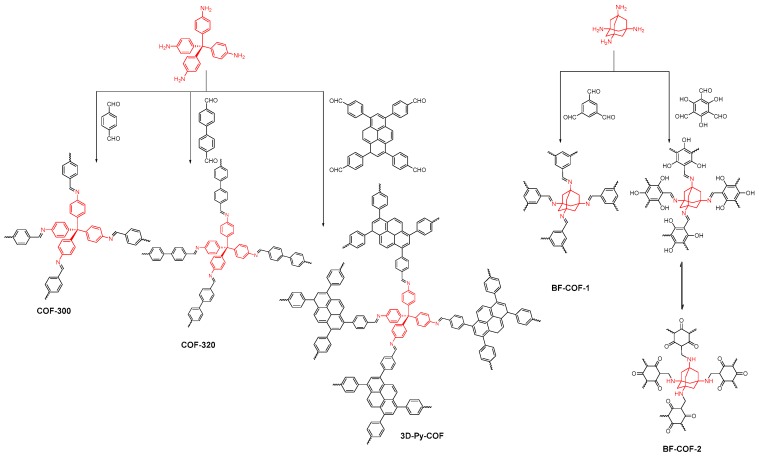
Construction of imine-linked COFs by co-condensation of tetratopic anilines (tetra-(4-anilyl)methane, and 1,3,5,7-tetraaminoadamantane) with various ditopic, tetraaldehydes and triangular aldehydes to form 3D networks.

To avoid the structural fragility of boron-containing COFs, imine-based COFs were discovered by a reversible condensation of polytopic anilines with polybenzaldehydes or polyketone with elimination of water by a catalyst of aqueous acetic acid (Figure 10). Generally, the crystallinity of the imine-based COFs is lower compared with that of boronate ester-linked COFs, whereas the chemical stability in the presence of water, acids and bases is significantly enhanced.

The first imine-linked COF was developed by Yaghi and co-workers in 2009 [25]. They constructed an imine-based COF (COF-300), which possesses a 3D five-fold interpenetrating diamond-like structure. COF-300 has a BET surface area of 1360 m^2^·g^−1^ and a pore size of 7.8 Å, which provides better hydrolytic stability compared to the boron-containing COFs. Moreover, the broad range of multifunctional amines and aldehydes provides a large number of structural possibilities for imine-based COFs.

Imine formed COF-320 was prepared through solvothermal condensation of tetra-(4-anilyl)-methane and 4,4′-biphenyldialdehyde in 1,4-dioxane at 120 °C. A 9-fold interlaced diamond network was formed and the final crystalline material exhibits high porosity with a Langmuir surface area of 2400 m^2^·g^−1^. The crystal structure of the COF-320 solid was elucidated by single-crystal 3D electron diffraction [26]. Recently, a pyridyl functionalized version of COF-320 was prepared, namely LZU-301, in which the reversible dynamic response upon guest accommodation and release was uncovered. Thus, the design principle of 3D dynamic COFs was claimed [27].

Wang and co-workers synthesized a novel 3D pyrene-based COF (3D-Py-COF) by condensation of tetra(*p*-aminophenyl)methane and 1,3,6,8-tetrakis(4-formylphenyl)-pyrene. This 3D-Py-COF has a two-fold interpenetrated **pts** topology, which was first reported in this work. Due to the existence of pyrene units in the 3D frameworks, 3D-Py-COF exhibits fluorescent properties and can be used in explosives detection [28].

Yan and co-workers designed and synthesized two new 3D base-functionalized COFs with micropores (BF-COF-1 and BF-COF-2), by the reaction of a tetrahedral alkyl amine, 1,3,5,7-tetraaminoadamantane (TAA), and 1,3,5-triformylbenzene (TFB) or triformylphloroglucinol (TFP) [29]. These BF-COFs were used for the Knoevenagel condensation reaction with high conversion (BF-COF-1: 96% and BF-COF-2: 98%), highly efficient size selectivity, and good recyclability. The structural rigidity of the admantane building unit is the key for forming targeted products. In contrast, the combination of tetra(*p*-aminophenyl) with TFB could not produce crystalline product.

**Figure 11:**
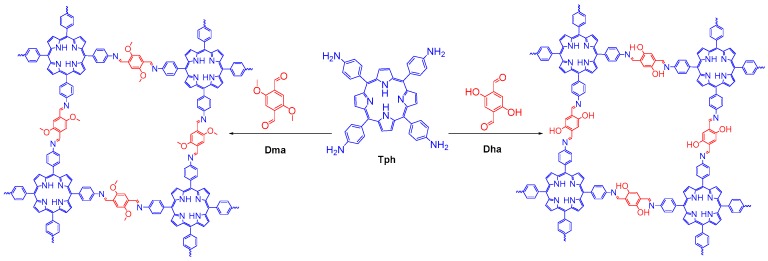
Syntheses of DmaTph and DhaTph by the condensation of square planar Tph building unit (blue) and linear Dma/Dha building unit (red). The introducing of -OH to the Schiff base [-C=N] centers in COFs can create an intramolecular [O-H···N=C] hydrogen bond to enhance both the chemical stability and crystallinity of COFs.

To enhance both the chemical stability and crystallinity in 2D porphyrin COFs, Banerjee and co-workers proposed a new strategy to protect the COF interior by introducing -OH to the Schiff base [-C=N] centers in COFs and creating an intramolecular [O-H···N=C] hydrogen bond (Figure 11) [30]. This hydrogen-bonding interaction enhances the stability of imine bonds in the presence of water and acid (3 N HCl). Otherwise, this hydrogen bond in DhaTph also could enhance the crystallinity and porosity, compared to the methoxy-substituted COF DmaTph in which this intramolecular hydrogen bond does not exist. This work demonstrates that the introduction of intramolecular hydrogen bonds can not only improve the chemical stability, but also the crystallinity and porosity of 2D COFs, which would broaden the potential applications of COF materials.

**Figure 12:**
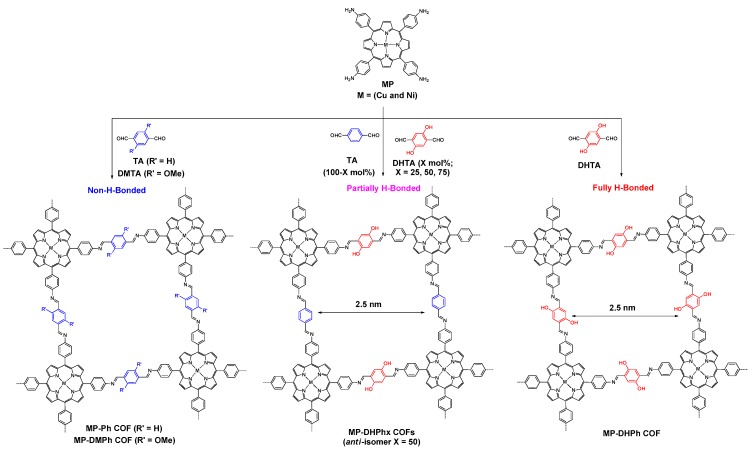
Schematic of the synthesis of 2D porphyrin COFs with designable content of hydrogen-bonding structures.

The formation of crystalline structures via relatively weak interactions between planar layers has been well defined and designed in crystal engineering. Jiang and co-workers synthesized a series of 2D COFs locked with intra-layer hydrogen-bonding (H-bonding) interactions (Figure 12) [31]. The H-bonding interaction sites were located on the edge units of the imine-linked tetragonal porphyrin COFs, and the contents of the H-bonding sites in the COFs were synthetically regulated by using a three-component condensation system. The intra-layer H-bonding interactions suppress the torsion of the edge units and lock the tetragonal sheets in a planar conformation. This planarization enhances the interlayer interactions and triggers extended π-cloud delocalization over the 2D sheets. Upon AA stacking, the resulting COFs with layered 2D sheets amplify these effects and strongly affect the physical properties of the material, including improving their crystallinity, enhancing their porosity, increasing their light-harvesting capability, reducing their band gap, and enhancing their photocatalytic activity toward the generation of singlet oxygen.

**Figure 13:**
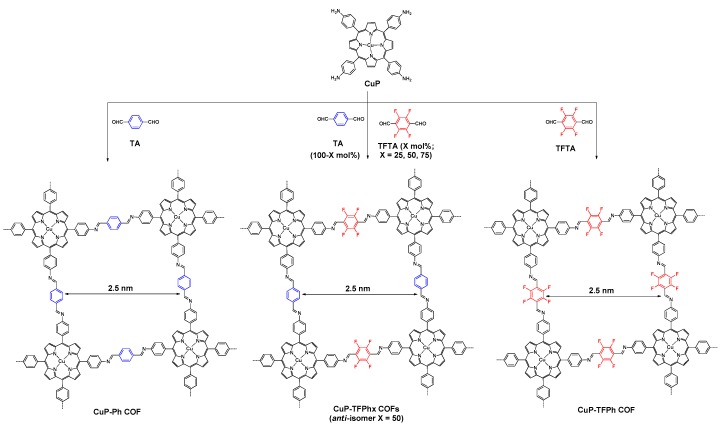
Schematic of the synthesis of 2D COFs integrated with self-complementary π-electronic forces (CuP−TFPh*_X_*, *X* = 25, 50, and 75 mol %) and the CuP−Ph and CuP−TFPh controls.

In addition to H-bonding interactions, self-complementary π-electronic forces also provide new opportunities for enhancing the crystallinity of COFs. Jiang and co-workers reported a synthetic method to control the crystallinity and porosity of COFs by managing interlayer interactions based on self-complementary π-electronic forces (Figure 13).

They controlled the crystal structure of CuP-Ph COF by introducing 2,3,5,6-tetrafluoroterephthalaldehyde into the COF structure. Fluoro-substituted aromatic units in different ratios were integrated into the edge units, which will induce the self-complementary π-electronic interactions in the COFs. This interaction increases the crystallinity and the porosity of COFs by maximizing the total crystal stacking energy and minimizing the unit cell size. This work provides a new pathway to improve the crystals of COFs by controlling the interlayer interactions [32]. 

**Figure 14:**
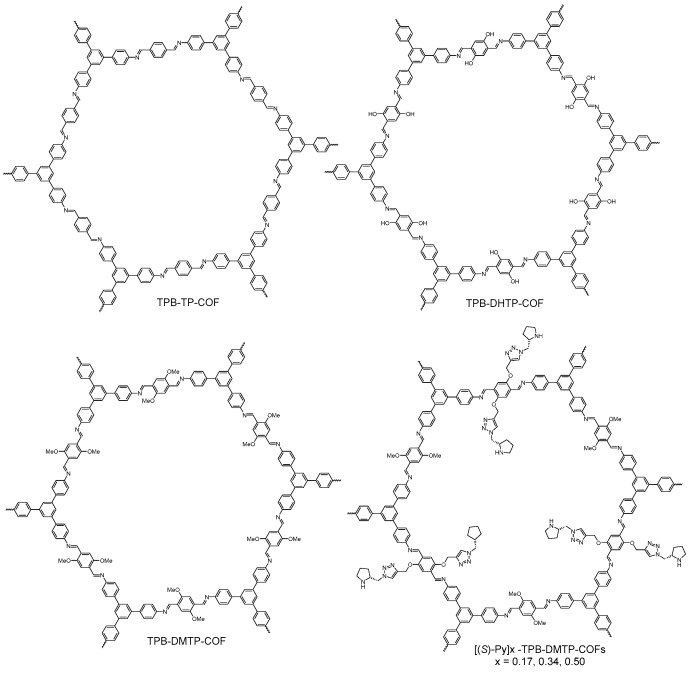
Synthesis and structure of stable crystalline porous COFs through softened interlayer charge repulsion. (**a**) TPB-TP COF control; (**b**) TPB-DHTP COF control; (**c**) stable crystalline porous COF of TPB-DMTP COF; (**d**) functionalization of TPB-DMTP COF ([(*S*)-Py]*x* -TPB-DMTP-COFs, *x* = 0.17, 0.34, 0.50).

**Figure 15:**
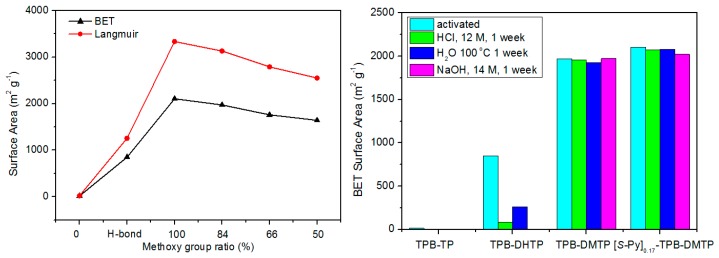
The surface area vs the methoxy group ratio in TPB-DMTP-COF (left). The BET surface areas of TPB-DMTP and (S)-Py-TPB-DMTP COFs use TPB-TP COF and TPB-DHTP COF as control.

The stability and crystallinity of 2D COFs are highly related to the factors of the interlayer interactions between the 2D layers. In imine-based COFs, the C=N bond is polarized to yield partially positively charged carbon and negatively charged nitrogen, which will cause electrostatic repulsion between the layers and could destabilize the layered structure staking. Jiang and co-workers introduced methoxy groups (-OMe) into the pore walls to reinforce the interlayer interactions (Figure 14). The oxygen lone pairs on the methoxy-substituted *C*_2_-phenyl edges soften the polarization through resonance and increase the stability and high crystallinity of the COF. Compared to the TPB-TP-COF and TPB-DHTP-COF, the methoxy-functionalized TPB-DMTP-COF has much higher crystal stacking energy, showing high crystallinity and high BET surface area. Through the introduction of chiral centers, (*S*)-Py moieties, onto the walls of the open channels, they obtained chiral COFs which can be used for heterogeneous asymmetric organocatalysis of asymmetric Michael reactions. The Jiang group also investigated the proton conduction properties of this highly crystalline and stable COF and explored the potential applications in many areas, including fuel cells, electrochemical sensors, electrochemical reactors and electrochromic devices [33].From Figure 15, we can observe that TPB-DMTP-COF also exhibits much higher BET and Langmuir surface area compared to the COFs without enhanced interlayer interactions and hydrogen bond enhanced COFs. As shown in Figure 15, the BET surface area decreases with the decrement of the methoxy group ratio, which demonstrates that the introduced methoxy groups greatly improve the crystallinity of the COFs. This kind of COF also shows enhanced chemical stability. After immersing in 12-M HCl, boiling water and 14-M NaOH for 1 week, the surface area of TPB-DMTP-COFs still retains the original values. However, the non-methoxy group-based COFs (TPB-TP-COF and TPB-DHTP-COF) show very week acid, base or boing water tolerance.

**Figure 16:**
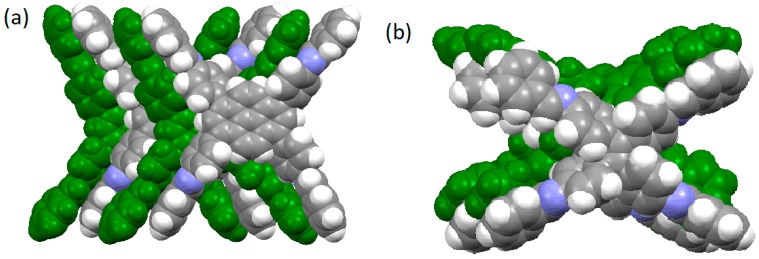
Different packing fashions between building units observed in the discrete model compounds of (**a**) 4PE-1P COF with 1,1,2,2-tetraphenylethene as the central unit and (**b**) Py-1P COF with 1,3,6,8-tetrakisphenylpyrene as the central unit, respectively.

Bein and co-workers found that a successive COF layer may nucleate at more than one location during the crystallizaation procedure, and the lateral offset of the growing pattern is more likely to be in various directions, which can induce lattice strain and defect into the crystals of COF materials (Figure 16). To prevent these defects from happening, they developed a synthetic concept to allow consecutive COF sheets to lock into position during crystal growth, and thus minimize the occurrence of stacking faults and dislocations. To prove their concept, they constructed a series of highly crystalline star-shaped, dual-pore 4PE-based COFs by co-condensation of 1,1,2,2-tetrakis(4-aminophenyl)ethane with the linear dialdehyde bridging units 1P, 2P, 3P and TT. They also synthesized a set of hexagonal 3PA- and 3PB-based COFs by combination of the linear dialdehyde bridging units 2P and TT as control COFs [34]. This approach enabled the achievement of a very high crystallinity for a series of COFs that comprise tri- and tetradentate central building blocks. Bein and co-workers also proposed that the molecular geometry of the central building block in COFs provides a uniquely defined docking site that can lead the attachment of successive COF layers, which will further enhance the crystallinity of the COFs [35]. They chose 1,3,6,8-tetrakis(4-aminophenyl)pyrene as a central building block to synthesize a series of highly crystalline pyrene-based COFs. As shown in Figure 16, the single-crystal structures of their molecular model compounds also revealed different packing preferences for different molecules.

**Figure 17:**
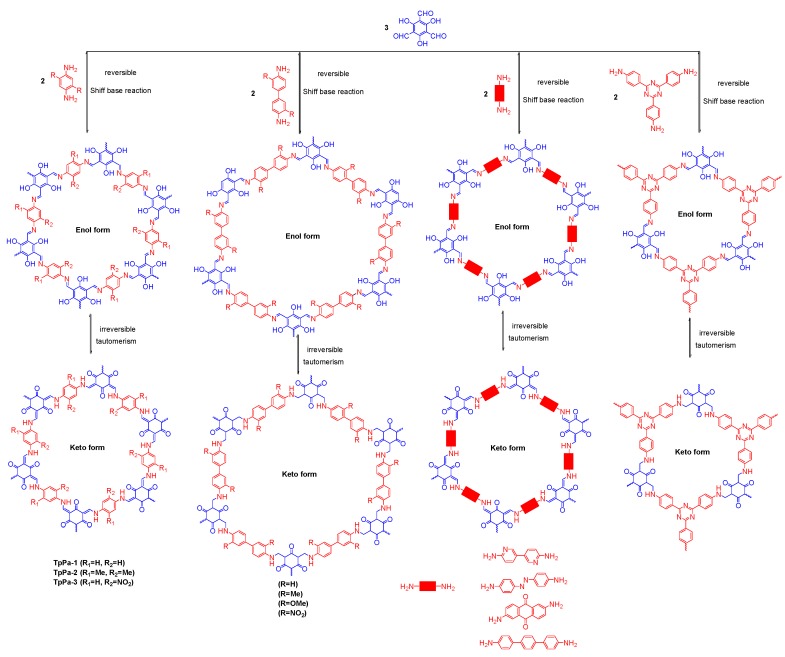
Construction of secondary amine-linked COFs via tautomerism by linking Tp with ditopic or tripic aniline-based linkers.

**Figure 18:**
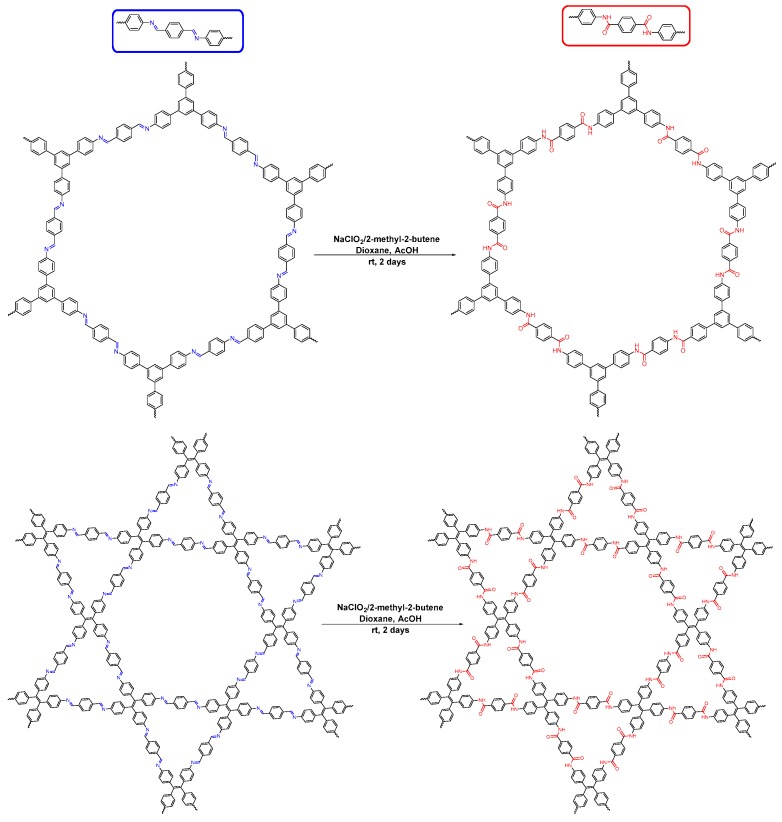
Construction of imide-linked COFs via post-oxidization of imine-linked COFs which bypass the difficulty of crystallization problem for irreversible imide bonds.

To achieve remarkable chemical stability, Banerjee and co-workers explored a new method to enhance the chemical stability of imine COFs by the transformation of combined reversible Schiff base bonds to irreversible β-ketoenamine bonds (Figure 17) [36]. They synthesized the COF TpPa-1 and TpPa-2 by the Schiff base reactions of 1,3,5-triformylphloroglucinol (Tp) with *p*-phenylenediamine (Pa-1) and 2,5-dimethyl-*p*-phenylenediamine (Pa-2), respectively. The expected enol-imine (OH) form underwent irreversible proton tautomerization into irreversible keto-enamine form, which confers outstanding stability to boiling water, aqueous acid (9 N HCl) and base (9 N NaOH). This linkage has also been synthesized by a simple, solvent-free, room-temperature mechanochemical synthetic route [37].The amide-linked COFs show improved chemical stability relative to their imine progenitors, but low degree of crystallinity. Recently, Yaghi and co-workers reported a chemical conversion of imine bond to amide bond inside COFs to improve both the chemical stability (including base and acid stability) and crystallinity (Figure 18). They constructed two layered imine COFs, TPB-TP-COF (**1**) and 4PE-1P-COF **(2**) as starting materials and then converted the imine-linked COFs to the amide-linked COFs, [C_6_H_3_(C_6_H_4_NH)_3_]_2_[C_6_H_4_(CO)_2_]_3_, **1**’ and [(C_6_H_4_NH)_4_][C_6_H_4_(CO)_2_]_2_, **2**’ without losing their crystallinity or topology. FT-IR and ^13^C CP-MAS NMR spectra indicated the successful conversion of COF **1** and COF **2** to COF **1**’ and COF **2**’. PXRD results demonstrated the high crystal structure of COF **1**’ and COF **2**’. The reduction of BET surface area of COF **1**’ and COF **2**’ compared to COF **1** and COF **2** due to the increase of framework mass and decrease of pore volume. This method offers a new pathway to overcome the usual crystallization problem in COF chemistry [38].

#### 2.2.2. Hydrazone-Linked COFs

**Figure 19:**
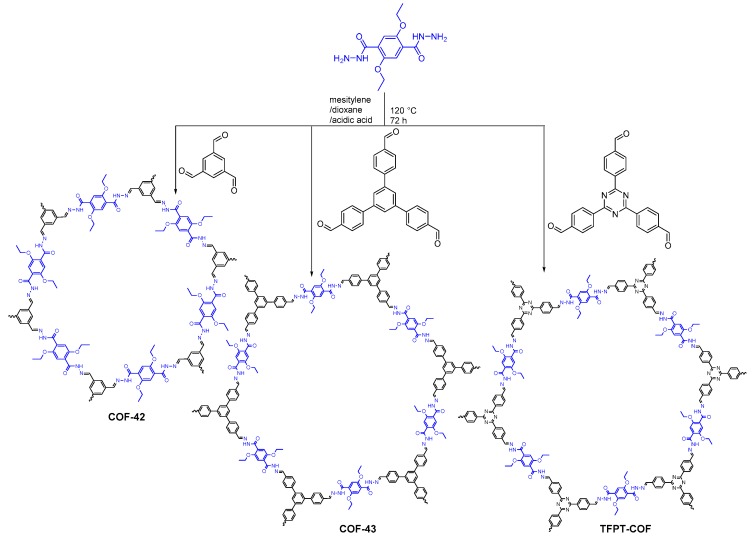
Synthetic reaction of hydrazone-linked COFs (COF-42, COF-43, and TFPT-COF).

Like the reactions of imine-based COFs, the reaction of benzaldehydes and hydrazide groups could yield hydrazone-linked COFs (Figure 19). Hydrazone-linked COFs show good chemical stability relative to imine-based ones due to the hydrogen-bonding interactions between the oxygen atoms in the alkoxyl chains and the hydrogens in -CONH- units. However, the functional monomers available for construction of hydrazone-based COFs are limited. Yaghi and co-workers first condensed a di-functional acylhydrazide (2,5-diethoxyterephthalohydrazide) and trifunctional aldehydes (1,3,5-triformylbenzene or 1,3,5-tris(4-formylphenyl)benzene) to provide two crystalline mesoporous COFs, COF-42 and COF-43 [39].

Dichtel et al. reported the exfoliation of the COF-43 in various solvents to yield few-layer 2D polymers. The obtained exfoliated COF-43 polymers showed an apparent loss of crystallinity by PXRD, but no apparent changes in its covalent linkages and high aspect ratios [40].

Stegbauer et al. reported a hydrazone-based COF (TFTP-COF) capable of visible-light driven hydrogen generation with Pt as a proton reduction catalyst (PRC). The COF, constructed by 1,3,5-tris-(4-formyl-phenyl)triazine (TFPT) and 2,5-diethoxy-terephthalohydrazide (DETH) building blocks, shows a layered structure with a honeycomb-type lattice featuring mesopores of 3.8 nm and gives a BET surface area of 1603 m^2^·g^−1^. When illuminated with visible light, the Pt-doped COF continuously produces hydrogen from water without signs of degradation. This new application of COFs in photocatalysis to open new pathways for heterogeneous photocatalysts [41].

Ding et al. rationally designed a thioether-based hydrazone-linked COF-LZU8, and use it for highly sensitive detection and effective removal of Hg^2+^. Due to the high stability of the hydrazone linkages, together with the dense distribution of the thioether groups and the straight channels in COF-LZU8, the recycling of COF-LZU8 achieved the simultaneous detection and removal of the toxic Hg^2+^ [42].

#### 2.2.3. Azine-Linked COFs

**Figure 20:**
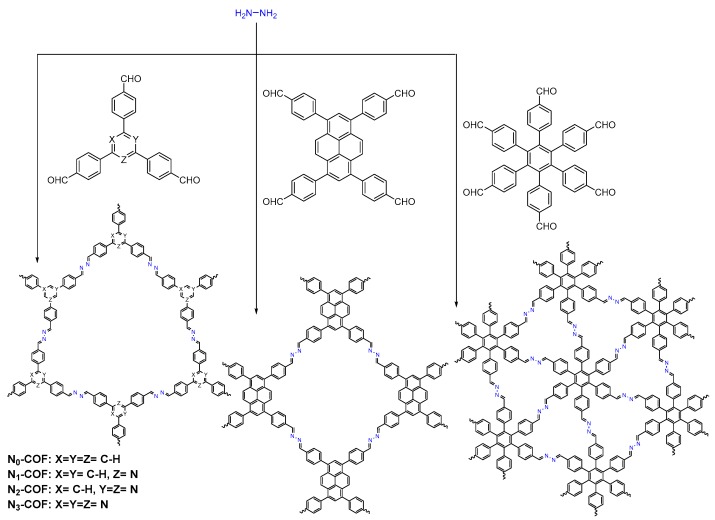
Synthetic reaction of azine-linked COFs (Py-Azine COF, HEX-COF 1, and N*_x_*-COF).

Azines (−C=N−N=C−) are formed by condensation of hydrazine with aldehydes (Figure 20). Azine-linked COFs usually have small micropores and are stable in water, acid and base. Dalapati et al. constructed a highly crystalline two-dimensional the Py-Azine COF by condensation of hydrazine with 1,3,6,8-tetrakis(4-formylphenyl)pyrene (TFPPy) under solvothermal conditions [43]. The azines inside of Py-Azine COF serve as Lewis basic sites to bind guest and offers a selective detection of 2,4,6-trinitrophenol (TNP), which exhibits a potential application for chemosensing systems. Li et al. synthesized an azine-linked covalent organic framework, ACOF-1, by condensation of hydrazine hydrate and 1,3,5-triformylbenzene. The high surface areas (1318 m^2^·g^−1^) and small pore size make it useful as a gas storage medium for CO_2_ (177 mg·g^−1^ at 273 K and 1 bar), H_2_ (9.9mg·g^−1^ at 273 K and 1 bar), CH_4_ (11.5 mg·g^−1^ at 273 K and 1 bar) [44,45]. Smaldone and co-workers developed a novel azine-linked hexaphenylbenzene-based covalent organic framework, HEX-COF 1, which shows excellent sorption capability for carbon dioxide (20 wt %) and methane (2.3 wt %) at 273 K and 1 atm [46]. Lotsch et al. reported a tunable water- and photostable azine COF by hydrazine and triphenylarene aldehydes for visible light induced hydrogen generation [47]. Zhang et al. reported an azo-containing COF by introducing a novel azobenzene monomer through the borate ester formation reaction of azobenzene-4,4′-diboronic acid (ABDA) and 2,3,6,7,10,11-hexahydroxytriphenylene (HHTP). The Azo-COF has a hexagonal skeleton and permanent porosity. The azo units in azo-COFs endow the COF material with photoisomerization properties [48].

#### 2.2.4. Squaraine-Linked COF

**Figure 21:**
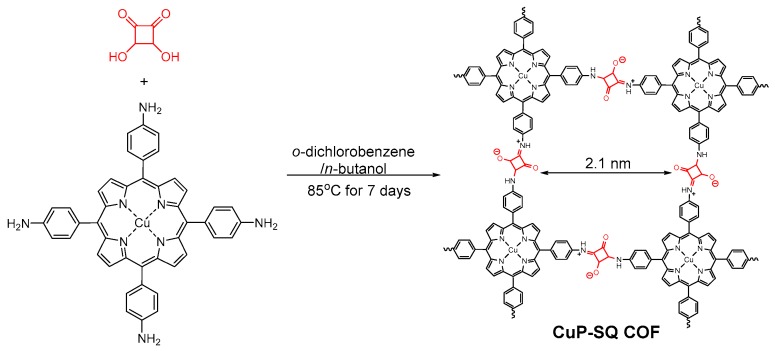
Synthetic reaction of squaraine-linked COFs (CuP-SQ COF).

The use of squaric acids and amines or hydrazines as building blocks has been developed for the synthesis of squaraine-linked COFs that allow the integration of zwitterion structures into the skeletons. Nagai et al. reported a new reaction based on squaraine for the synthesis of a new type of squaraine-based 2D COF. They used copper(II) 5,10,15,20-tetrakis(4-aminophenyl)porphyrin (TAP-CuP) as a building block to construct a crystalline 2D conjugated COF (CuP-SQ COF) with a tetragonal mesoporous skeleton (Figure 21). The CuP-SQ COF has a BET surface area of 2289 m^2^·g^−1^ with a pore size of 2.1 nm. This research expands the types of COFs. Zhang and their co-workers constructed a novel functional covalent organic framework by the porphyrin building block (meso-tetra-4-hydrazidocarbonylphenyl) porphyrin, THCPP) and squaric acid via a bottom-up approach. The reported covalent porphyrin framework with coordinated manganese (III) ions (Mn–CPF-2) presents promising catalytic properties for the selective oxidation of olefins [49].

#### 2.2.5. Imide-Linked COFs

**Figure 22:**
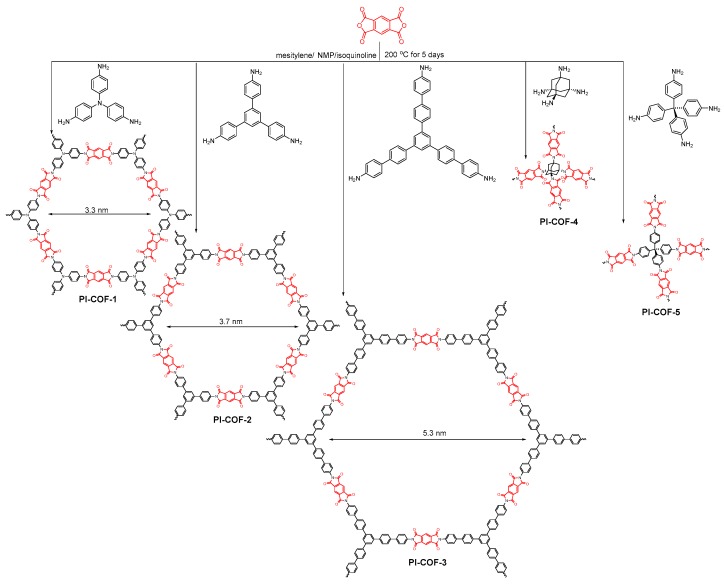
Construction of imide-linked COFs by stitching tritopic or tetratopic polyaniline with benzene-1,2,4,5-tetracarboxylic dianhydride into 2D and 3D networks.

Polyimides are formed by condensing primary amines and anhydrides (Figure 22). Yan and co-workers synthesized three-dimensional porous crystalline polyimide covalent organic frameworks (PI-COFs) by choosing tetrahedral building units of different sizes [50].

These PI-COFs show high thermal stability (>450 °C) and surface area (up to 2403 m^2^·g^−1^), as well as narrow pore size distribution (13 Å for PI-COF-4 and 10 Å for PI-COF-5). Due to the high surface area and large pore size, they were the first to be employed in controlled drug delivery, where they show high loadings and good release control for ibuprofen (IBU). These results provide a pathway of COFs for pharmaceutical applications. The Yan group also synthesized a series of polyimide-based COFs with different pore size by changing the length of tri-amines, using the imidization reaction [51]. Among them, the PI-COF-3, synthesized by condensation of PMDA and the extended triamine 1,3,5-tris[4-amino(1,1-biphenyl-4-yl)]benzene (TABPB) has 5.3 nm wide pores and were loaded with Rhodamine B, a dye probe for biological applications. The dye-doped PI-COF-3 exhibits clear temperature-dependent photoluminescence, which facilitate the development of dye-doped porous materials for applications in temperature-sensing devices.

#### 2.2.6. Phenazine-Linked COFs

**Figure 23:**
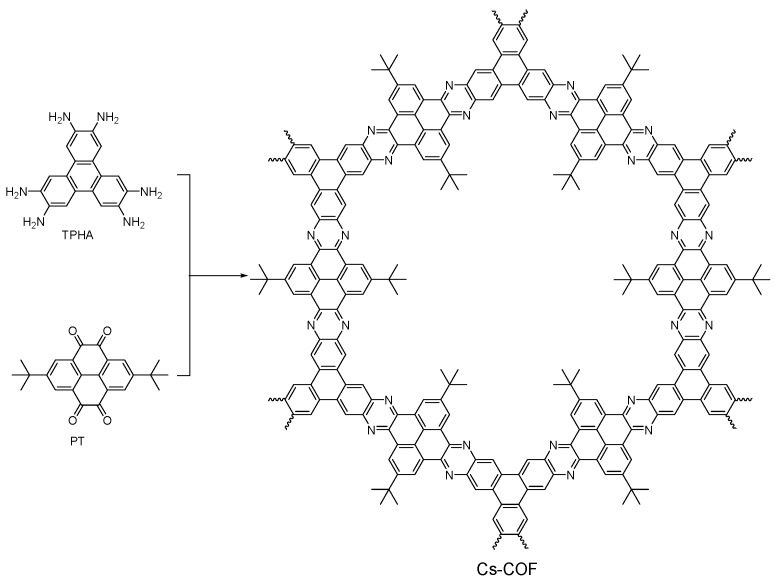
Synthetic reaction of the phenazine-linked COF.

Aza-fused linkages were pursued in making conductive organic materials that have many potential applications in electrochemical devices [52]. However, the great challenge is how to make them highly crystalline. Jiang and co-workers prepared a phenazine-linked COF (CS-COF, Figure 23) through ring-fusing reactions between quinone and amine derivatives catalyzed by acidic forms. They used C_3_-symmetric triphenylene hexamine (TPHA) and C_2_-symmetric tertbutylpyrene tetraone (PT) as building blocks to prepare the crystalline phenazine-linked CS-COF under solvothermal conditions. The polygon skeletons of CS-COF are highly π conjugated and chemically stable in various solvents [53].

#### 2.2.7. Triazine-Linked COFs

**Figure 24:**
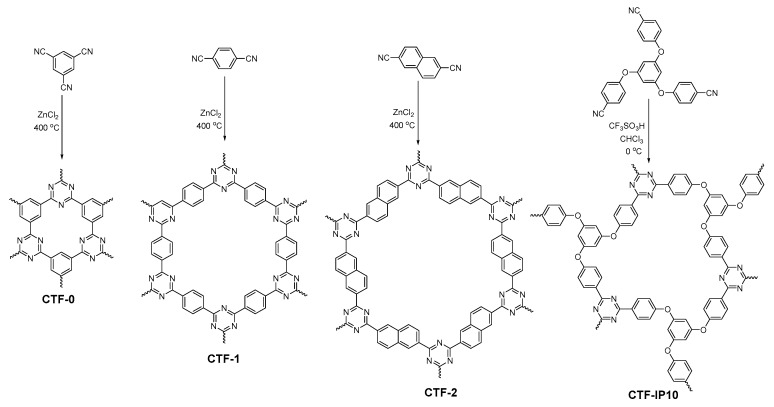
Construction of crystalline covalent triazine frameworks by self-condensation of aromatic nitriles.

The self-condensation of aromatic nitriles at high temperature in molten salts or at room temperature in the presence of a strong acid catalyst, such as trifluoromethylsulfonic acid, can form crystalline, porous, covalent triazine frameworks (CTFs, Figure 24). The first CTF was reported by Thomas and co-workers in 2008, and is synthesized through the dynamic trimerization of aromatic nitriles (1,3,5-triazines) using a ZnCl_2_ ionothermal method [54]. CTFs provide high surface area (up to 3000 m^2^·g^−1^), low densities, and outstanding thermal/chemical stability. However, CTFs exhibit short-range crystalline order and limited pore size distributions in some cases [55].

Some CTFs were prepared in molten ZnCl_2_ at high reaction temperatures (400 °C~700 °C). Yet these conditions for CTF preparation obtained less crystalline CTFs of limited functionality, and only from 1,4-dicyanobenzene and 2,6-dicyanonaphthalene [56]. Then, Cooper et al. [57] developed a microwave-assisted, acid-catalyzed method to synthesize triazine networks with fewer discolored impurities (P1M-P6M). Their structural and synthesized tunability developed their specific applications, such as gas storage, gas separation, catalyst support, and organic dye separation.

Thomas et al. synthesized a covalent triazine framework (CTF-0) by trimerization of 1,3,5-tricyanobenzene in molten ZnCl_2_ [58]. This material showed high CO_2_ uptakes and good catalytic activity for CO_2_ cycloaddition. The high surface area and robust porous structures of the organic framework were explored for highly selective gas capture.

Ghosh et al. explored a strategy to obtain a porous covalent triazine framework (CTF-IP-10) with bimodal functionality by acid-catalysed room temperature reaction of a tricyanomonomer (PCN-M1) in CHCl_3_ [59]. The obtained CTF-IP 10 consists both an electron-deficient central triazine core and electron-rich aromatic building blocks.

### 2.3. Hetero Linkages in One COF Structure

To improve the functionality and stability of imine-based COF structures, design and construction of COFs connected by two or more types of covalent bonds based on orthogonal reactions were developed. 

**Figure 25:**
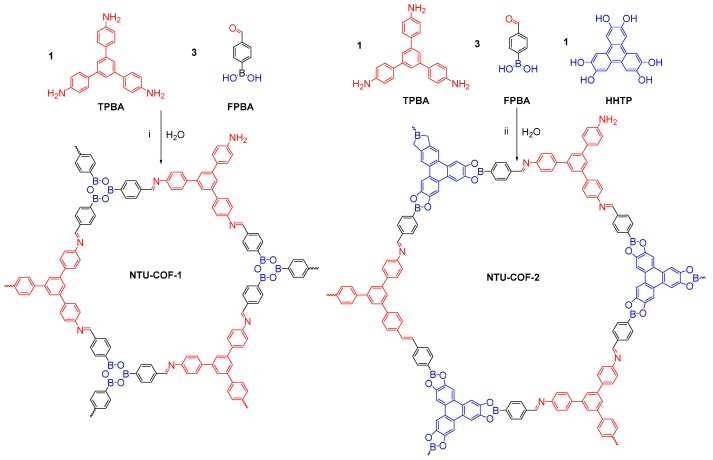
Syntheses of (i) NTU-COF-1 and (ii) NTU-COF-2 by two types of covalent bonds from orthogonal reactions.

First the Zhao group constructed a 2D COF (NTU-COF-1, Figure 25) by the formation of imine groups and B_3_O_3_ boroxine rings, involving the utilization of two building blocks: 4-formyl-phenylboronic acid (FPBA) and 1,3,5-tris(4-aminophenyl)-benzene (TAPB). They also constructed a ternary COF (NTU-COF-2, Figure 25) through the formation of imine groups and C_2_O_2_B boronate rings from FPBA, TAPB and HHTP, which involved the formation of two types of covalent bonds. The NTU-COF-2 possesses high BET surface areas of 1619 m^2^·g^−1^ and mesopores with a diameter of 2.6 nm. The design and construction of COFs by this orthogonal reaction strategy is a promising approach in rational COF development [60].

**Figure 26:**
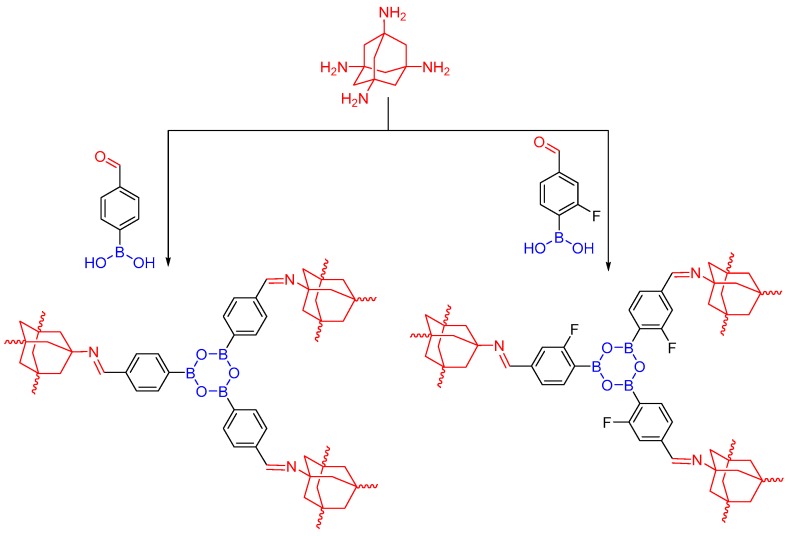
Syntheses 3D COFs of (i) DL-COF-1 and (ii) DL-COF-2 by two types of covalent bonds from orthogonal reactions.

The Qiu group synthesized two extended 3D COFs (DL-COF-1, DL-COF-2) with dual linkages, imine groups and B_3_O_3_ boroxine rings (Figure 26), involving the utilization of two 1,3,5,7-tetraaminoadamantane (TAA) and 4-formylphenylboronic acid (FPBA) or 2-fluoro-4-formylphenylboronic acid (FFPBA) building blocks [61]. The obtained DL-COF-1 and DL-COF-2 showed high BET surface areas of 2259 m^2^·g^−1^ and 2071 m^2^·g^−1^, respectively.

## 3. Gas Storage for Clean Energy Applications

COFs have been touted as a new class of porous crystalline materials for gas storage applications due to their high inherent surface area, very low densities (i.e., being made of the elements H, C, N, B, O, Si), high stability (formation of covalent bonds), and tunable pore dimensions (from ultramicropores to mesopores). The gas storage capacity of COFs for hydrogen, methane, and carbon dioxide, has attracted increasing interest in the past decades [62].

### 3.1. Hydrogen Storage

The demand for the reduction of air pollution has led to the search of new and clean energy sources. Hydrogen gas storage is one of the bottleneck for the realization of an energy economy based on hydrogen’s high chemical abundance, being a clean energy carrier, and high chemical energy density. The demand of the US Department of Energy (DOE) for hydrogen storage is 5.5 wt % in gravimetric capacity, and 40 kg·m^−3^ of volumetric capacity at operating temperature of 233–333 K with a pressure of 100 atm by the year 2017. Recently, a large number of porous materials have been explored for the application of hydrogen storage. However, none of the candidate materials developed so far has satisfied the DOE target. COF materials, as new crystal porous materials, have also attracted increasing interest in hydrogen storage applications.

**Figure 27:**
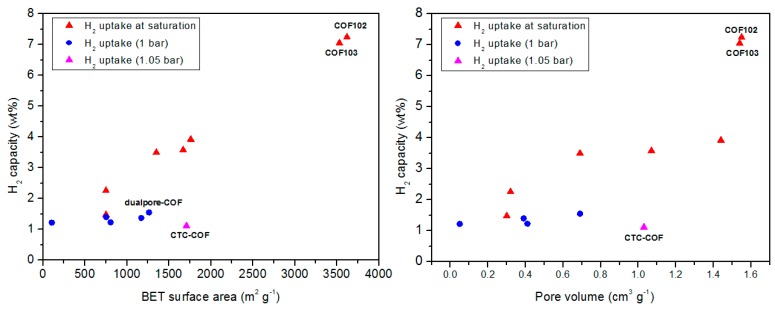
Plot of H_2_ uptake against BET surface area (**left**) and pore volume (**right**) for the selected COFs at 77 K.

Figure 27 and Table 1 show the capabilities of hydrogen storage reported for different COF materials. The plot of hydrogen uptake against surface area and pore volume for the selected COFs at 77 K are shown in Table 1. COF-18Å (S_BET_: 1263 m^2^·g^−1^, pore volume: 0.65 cm^3^·g^−1^, pore size: 1.8 nm) showed the highest hydrogen uptake at low pressure (1 bar and 77 K) among the similar 2D COFs with different alkyl chain lengths (compared to COF-11Å, COF-14Å, and COF-16Å). For context, 3D COFs (COF-102 and COF-103) show much higher hydrogen storage capacities due to the higher surface areas and lower densities, compared to 2D COFs. Among the different COF materials for H_2_ uptake, the one with the largest storage capacity is 3D COF-102 (Table 1, S_BET_: 3620 m^2^·g^−1^, pore volume: 1.55 cm^3^·g^−1^, pore size: 1.2 nm), which uptakes 72 mg per gram at 1 bar and 77 K. This capacity is comparable to those of MOF-177 (75 mg·g^−1^, S_BET_: 4500 m^2^·g^−1^), MOF-5 (76 mg·g^−1^, S_BET_: 3800 m^2^·g^−1^), and the porous aromatic framework PAF-1 (75 mg·g^−1^, S_BET_: 5600 m^2^·g^−1^). These capacities significantly demonstrate the potential of COFs for use as hydrogen storage materials.

From the summarized H_2_ storage results in Figure 27, we find that the high-pressure H_2_ adsorption of COFs with higher surface areas and larger pore possess higher hydrogen uptake capacities when measured under the same conditions. Meanwhile, the COFs with higher pore volume show higher hydrogen uptake. These results demonstrate that the high-pressure H_2_ uptake capacities are dominantly determined by pore volumes and S_BET_ of COFs. The H_2_ uptake capacity at 77 K and 1 bar increased in the order of COF-11Å < COF-14Å < COF-16Å < COF-18Å, revealing that the low-pressure H_2_ uptake also depends on the pore size of COFs.

However, the hydrogen storage of COFs at ambient pressure and temperature is still low and far from meeting the DOE requirements. Adsorption simulations of the COFs show that doping with charged species can affect their capacity. Theoretical studies [63,64] show that metal-doped COFs can enhance the hydrogen storage at a temperature range of 273 to 298 K. Recently, scientists have theoretically predicted that lithium doped COFs would exhibit improved hydrogen storage capacity due to the increased binding energy between the H_2_ and the Li atom. These simulation studies predict that the hydrogen uptakes for Li-doped COF-105 and COF-108 (6.84 and 6.73 wt %, respectively, at 298 K and 100 bar) excelled beyond the non-doped COFs and the previous reported MOF materials. Although the experimental and theoretical results indicate that COFs are promising candidates for hydrogen storage, the practical and industrial use of COF materials toward the DOE target for hydrogen uptakes is still far away.

### 3.2. Methane Storage

Methane, as an abundant and inexpensive gas, stands out as a potential vehicular fuel to replace conventional fossil fuels. According to the US DOE requirement, the current target for methane storage is 350 cm^3^_STP_ cm^−3^_adsorbent_ (*v*/*v*) and 0.5 g_CH4_ g_adsorbent_^−1^ (699 cm^3^_STP_ g^−1^) at 35 bar and 298 K (STP: standard temperature and pressure). From Table 2, the 2D COF, COF-1 shows CH_4_ uptake of 40 mg·g^−1^ at 298 K, 35 bar. However, upon increasing the pressure further, no increase of methane adsorption is observed due to the small pores in COF-1. The pores in COF-1 are quickly occupied by CH_4_ molecules at low pressures and higher pressures may not compact the gas any more. The largest pore, 3D COFs, COF-102 shows the highest observed CH_4_ storage capacity (187 mg·g^−1^). COF-103 also possesses a high methane capacity of 175 mg·g^−1^. These values are comparable to the highest methane uptake for MOFs (MOF-210; 220 mg·g^−1^).Theoretical simulation studies indicate that lithium doped COFs could significantly strengthen the binding between methane and Li^+^ cations, which will further improve the methane capacity of COFs. The results show that the methane storage of lithium doped COFs are double at 298 K and low pressures (< 50 bar), compared to the non-doped COFs.

The abovementioned studies indicate the potential application of COFs for methane storage at ambient temperatures and 35 bar. However, the practical use of COF materials to meet the requirement of DOE target for is a big challenge.

### 3.3. CO_2_ Capture

The rise of carbon dioxide is one of the global issues, which is causing global warming, rising sea levels, and the increasing acidity of the oceans. The development of effective strategies for capturing CO_2_ has attracted broad interest. Among the current strategies, capture of CO_2_ by crystal porous materials is energetically efficient and technically feasible. Yaghi and co-workers first studied a series of 2D COFs with 1D micropores (COF-1 and COF-6), 2D COFs with 1D mesopores (COF-5, COF-8 and COF-10), and 3D COFs with 3D medium sized pores (COF-102 and COF-103) on application of CO_2_ capture in 2009. From Table 3, we can observe that the storage of CO_2_ at 298 K and 55 bar, follow the order of COF-102 > COF-103 > COF-10 > COF-5 > COF-8 > COF-6 > COF-1, which indicates that the high-pressure CO_2_ capture are highly related to the pore volumes and SA_BET_ of COFs.

Functional groups inside the channel walls of COF materials can enhance the CO_2_ adsorption and separation. Fluorinated alkanes exhibit an extraordinary affinity to CO_2_ molecules. Han et al. reported a fluorinated triazine-based COF (FCFT-1 and FCTF-1-600) for CO_2_ capture by using tetrafluoroterephthalonitrile as a monomer [65]. The triazine-based COFs haveN-rich frameworks, which are favored for CO_2_ adsorption, meanwhile, the high electronegativity of F enhances CO_2_ electrostatic interaction. The CO_2_ adsorption of these triazine-based COF materials at 273 K and 1 bar were as follows, FCTF-1-600 (124 cm^3^·g^−1^) > FCTF-1 (105 cm^3^·g^−1^) > CTF-1-600 (86 cm^3^·g^−1^) > CTF-1 (55 cm^3^·g^−1^). These results demonstrate that the fluorizated COF materials exhibit much better CO_2_ uptake under low pressure. Jiang et al. converted a conventional hydroxyl group modified 2D COF ([HO]_x%_-H_2_P-COFs) into an outstanding carbondioxide-capture material, carboxyl group modified 2D COF ([HO_2_C]*_x%_*-H_2_P-COFs) [64]. The [HO_2_C]_x%_-H_2_P-COFs show micropores between 1.4-2.2 nm instead of the mesopores of [HO]*_x%_*-H_2_P-COFs (2.5 nm). From the results of Table 3, the [HO]*_x%_*-H_2_P-COFs have low CO_2_ capacity between 23-32 cm^3^·g^−1^ at 273 K and 1 bar. However, [HO_2_C]*_x%_*-H_2_P-COFs exhibit a dramatically enhanced CO_2_ adsorption between 49–89 m^3^·g^−1^ under the same conditions.

**Figure 28:**
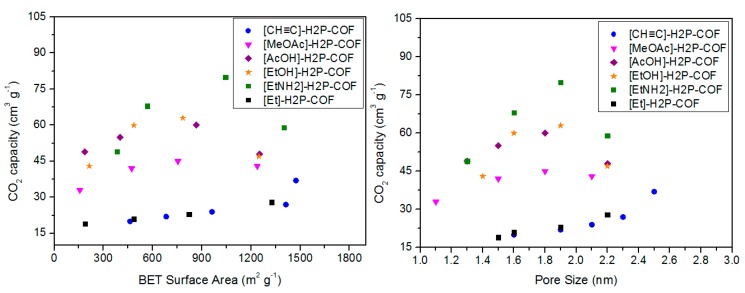
Plot of low-pressure CO_2_ uptake against BET surface area (**left**) and pore size (**right**) for the selected COFs at 273 K and 1 bar.

Jiang et al. also carried out a similar strategy to modify the pore surface with different functional groups through click reactions [66,67]. From Table 3, we can observe that different function of pore walls of COF materials can induce the decrease of surface area, pore sizes and pore volumes. However, the capacity for CO_2_ adsorption was highly dependent on the structures of the functional groups. Both from Table 3 and Figure 28, ethyl functionalized COFs, [Et]*_x_*-H_2_P-COFs, exhibit similar CO_2_ adsorption capacity to those of [CH≡C]_x_-H_2_P-COFs. The CO_2_ adsorption capacities of [CH≡C]*_x_*-H_2_P-COFs and [EtNH_2_]*_x_*-H_2_P-COFs enhanced with the increase of BET surface area and pore size. However, [MeOAc]*_x_*-H_2_P-COFs, [AcOH]*_x_*-H_2_P-COFs, [EtOH]*_x_*-H_2_P-COFs and [EtNH_2_]*_x_*-H_2_P-COFs show much higher CO_2_ adsorption capacity than those of [CH≡C]*_x_*-H_2_P-COFs. The CO_2_ adsorption capacities are in order of [CH≡C]*_x_*-H_2_P-COFs ≈ [Et]*_x_*-H_2_P-COFs ≪ [MeOAc]*_x_*-H_2_P-COFs < [AcOH]*_x_*-H_2_P-COFs ≈ [EtOH]*_x_*-H_2_P-COFs < [EtNH_2_]*_x_*-H_2_P-COFs, which is due to the interaction between the functional group and CO_2_ molecule. These results indicate that the CO_2_ adsorption performances are highly related to the pore surface of the COF materials.

## 4. Perspectives and Challenges

The past decade has been witnessed the blooming COF chemistry and their applications. Their unique features, such as flexible molecular structure design, permanent porosity, low density, modest to high crystallinity and controllable pore size from ultraporous to mesoporous, and the diversity of available building blocks, easily make them targets for structural and functional design, and promise their great potential as materials for application in various areas. However, there are still a lot of challenges in the design and synthesis of COFs.

First, compared to the high ordered structure of MOF materials, further investigation on the control of crystal growth, the skeletons, and pores is still of great importance. Currently, thermodynamically controlled solvothermal reactions are widely used to synthesize COF materials. The investigations reveal that the formation and growth of crystal nuclei to improve the crystallinity of COFs is quite important. Such studies provide a general pathway for the preparation of high crystallinity COFs. Moreover, control over the defect sites, the morphology and the stacking mode of COF’s structures are also important and effective methods to construct precise crystal COF structures are necessary. At present, no direct analytical tools can be used to visualize and analyze the stacking and topological structures of COFs. Thus, the creation of single-crystal COFs an unattainable target.

Meanwhile, through the existing approaches, the methods to increase the surface area and porosities of COF materials are a big bottleneck. The surface area and porosities are the most important parameters for COF materials for gas adsorption and gas separation applications. How to design COFs structures which can improve both surface area and these properties is the key to realize the potential application of COFs in these fields. For the 2D COFs, spatially enlarging the interlayered spaces would increase the surface areas. For 3D COFs, the construction of highly crystalline frameworks would lead to very high BET surface areas that can reach the theoretical *S*_BET_ over 6000 m^2^·g^−1^.

## Figures and Tables

**Table 1:** BET surface area, porosity parameters and H_2_ capture of selected COFs.

COF Materials	BET Surface Area (m^2^·g^−1^)	Pore Size (Å)	Pore Volume (V_p_, cm^3^·g^−1^)	H_2_ Uptake (wt %)	Ref.
COF-1	750	9	0.3	1.48	[62]
COF-5	1670	27	1.07	3.58	
COF-6	750	6.4	0.32	2.26	
COF-8	1350	18.7	0.69	3.5	
COF-10	1760	34.1	1.44	3.92	
COF-102	3620	11.5	1.55	7.24	
COF-103	3530	12.5	1.54	7.05	
COF-1	628	9	0.36	1.28 (1 bar)	
COF-11Å	105	11	0.05	1.22 (1 bar)	
COF-14Å	805	14	0.41	1.23(1 bar)	
COF-16Å	753	16	0.39	1.4 (1 bar)	
COF-18Å	1263	18	0.69	1.55 (1 bar)	
CTC-COF	1710	22.6	1.03	1.12 (1.05 bar)	
dualpore-COF	1771	7.3, 25.2	3.189 (P/P° 0.99)	1.37 (1 bar)	

**Table 2:** BET surface area, porosity parameters and CH_4_ capture of selected COFs.

COF Materials	BET Surface Area (m^2^·g^−1^)	Pore Size (Å)	Pore Volume (V_p_, cm^3^·g^−1^)	CH_4_ Uptake (mg·g^−1^) *^a^*	Ref.
COF-1	750	9	0.3	40	[62]
COF-5	1670	27	1.07	89	
COF-6	750	6.4	0.32	65	
COF-8	1350	18.7	0.69	87	
COF-10	1760	34.1	1.44	80	
COF-102	3620	11.5	1.55	187	
COF-103	3530	12.5	1.54	175	
ILCOF-1	2723	23	1.21	129 (L·L^−1^)	

*^a^*CH_4_ uptake at 35 bar and 298 K.

**Table 3:** BET surface area, porosity parameters and CO_2_ capture of selected COFs.

COF Materials	BET Surface Area (m^2^·g^−1^)	Pore Size (nm)	Pore Volume (*V*_p_, cm^3^·g^−1^)	CO_2_ Uptake (cm^3^·g^−1^)		Ref.
				Low *^a^*	High *^b^*	
COF-1	750	0.9	0.3	51	117	[39]
COF-5	1670	2.7	1.07	31	443	
COF-6	750	0.64	0.32	85	158	
COF-8	1350	1.87	0.69	33	321	
COF-10	1760	3.41	1.44	27	514	
COF-102	3620	1.15	1.55	34	611	
COF-103	3530	1.25	1.54	38	606	
FCTF-1	662	0.46,0.54	-	105		[65]
FCTF-1-600	1535	0.46,0.59	-	124		
CTF-1	746	0.54		55		
CTF-1-600	1553	-		80		
[HO]_25%_-H_2_P-COF	1054	2.5		27		[66]
[HO]_50%_-H_2_P-COF	1089	2.5		23		
[HO]_75%_-H_2_P-COF	1153	2.5		26		
[HO]_100%_-H_2_P-COF	1284	2.5		32		
[HO_2_C]_25%_-H_2_P-COF	786	2.2		49		
[HO_2_C]_50%_-H_2_P-COF	673	1.9		68		
[HO_2_C]_75%_-H_2_P-COF	482	1.7		80		
[HO_2_C]_100%_-H_2_P-COF	364	1.4		89		
[CH≡C]_0_-H_2_P-COF	1474	2.5	0.75	37		[67]
[CH≡C]_25_-H_2_P-COF	1413	2.3	0.71	27		
[CH≡C]_50_-H_2_P-COF	962	2.1	0.57	24		
[CH≡C]_75_-H_2_P-COF	683	1.9	0.42	22		
[CH≡C]_100_-H_2_P-COF	462	1.6	0.28	20		
[Et]_25_-H_2_P-COF	1326	2.2	0.55	28		
[Et]_50_-H_2_P-COF	821	1.9	0.48	23		
[Et]_75_-H_2_P-COF	485	1.6	0.34	21		
[Et]_100_-H_2_P-COF	187	1.5	0.18	19		
[MeOAc]_25_-H_2_P-COF	1238	2.1	0.51	43		
[MeOAc]_50_-H_2_P-COF	754	1.8	0.42	45		
[MeOAc]_75_-H_2_P-COF	472	1.5	0.31	42		
[MeOAc]_100_-H_2_P-COF	156	1.1	0.14	33		
[AcOH]_25_-H_2_P-COF	1252	2.2	0.52	48		
[AcOH]_50_-H_2_P-COF	866	1.8	0.45	60		
[AcOH]_75_-H_2_P-COF	402	1.5	0.32	55		
[AcOH]_100_-H_2_P-COF	186	1.3	0.18	49		
[EtOH]_25_-H_2_P-COF	1248	2.2	0.56	47		
[EtOH]_50_-H_2_P-COF	784	1.9	0.43	63		
[EtOH]_75_-H_2_P-COF	486	1.6	0.36	60		
[EtOH]_100_-H_2_P-COF	214	1.4	0.19	43		
[EtNH_2_]_25_-H_2_P-COF	1402	2.2	0.58	59		
[EtNH_2_]_50_-H_2_P-COF	1044	1.9	0.5	80		
[EtNH_2_]_75_-H_2_P-COF	568	1.6	0.36	68		
[EtNH_2_]_100_-H_2_P-COF	382	1.3	0.21	49		

*^a^*CO_2_ uptake was measured at 1 bar and 273 K; *^b^*CO_2_ uptake was measured at 55 bar and 298 K.
